# Noble metal-modified titania with visible-light activity for the decomposition of microorganisms

**DOI:** 10.3762/bjnano.9.77

**Published:** 2018-03-07

**Authors:** Maya Endo, Zhishun Wei, Kunlei Wang, Baris Karabiyik, Kenta Yoshiiri, Paulina Rokicka, Bunsho Ohtani, Agata Markowska-Szczupak, Ewa Kowalska

**Affiliations:** 1 Institute for Catalysis, Hokkaido University, N21 W10, 001-0021 Sapporo, Japan; 2 School of Materials and Chemical Engineering, Hubei University of Technology, 430068 Wuhan, China; 3 Graduate School of Environmental Science, N10 W5, 060-0810 Sapporo, Japan; 4 Institute of Inorganic Technology and Environment Engineering, West Pomeranian University of Technology, Szczecin, Pulaskiego 10, 70-322 Szczecin, Poland

**Keywords:** antifungal properties, antimicrobial properties, bactericidal effect, noble-metal nanoparticles, plasmonic photocatalysis

## Abstract

Commercial titania photocatalysts were modified with silver and gold by photodeposition, and characterized by diffuse reflectance spectroscopy (DRS), X-ray powder diffraction (XRD), X-ray photoelectron spectroscopy (XPS) and scanning transmission electron microscopy (STEM). It was found that silver co-existed in zero valent (core) and oxidized (shell) forms, whereas gold was mainly zero valent. The obtained noble metal-modified samples were examined with regard to antibacterial (*Escherichia coli* (*E. coli*)) and antifungal (*Aspergillus niger* (*A. niger*), *Aspergillus melleus* (*A. melleus*), *Penicillium chrysogenum* (*P. chrysogenum*), *Candida albicans* (*C. albicans*)) activity under visible-light irradiation and in the dark using disk diffusion, suspension, colony growth (“poisoned food”) and sporulation methods. It was found that silver-modified titania, besides remarkably high antibacterial activity (inhibition of bacterial proliferation), could also decompose bacterial cells under visible-light irradiation, possibly due to an enhanced generation of reactive oxygen species and the intrinsic properties of silver. Gold-modified samples were almost inactive against bacteria in the dark, whereas significant bactericidal effect under visible-light irradiation suggested that the mechanism of bacteria inactivation was initiated by plasmonic excitation of titania by localized surface plasmon resonance of gold. The antifungal activity tests showed efficient suppression of mycelium growth by bare titania, and suppression of mycotoxin generation and sporulation by gold-modified titania. Although, the growth of fungi was hardly inhibited through disc diffusion (inhibition zones around discs), it indicates that gold does not penetrate into the media, and thus, a good stability of plasmonic photocatalysts has been confirmed. In summary, it was found that silver-modified titania showed superior antibacterial activity, whereas gold-modified samples were very active against fungi, suggesting that bimetallic photocatalysts containing both gold and silver should exhibit excellent antimicrobial properties.

## Introduction

Environmental pollution and the lack of clean potable water are main issues facing human development. Although, various methods of efficient control and monitoring of waste management have been successfully applied in developed countries, pollution in low-income countries is still a major environmental and public health challenge. It is thought that in developing countries, the quest for poverty reduction and economic development results in little consideration for environmental issues. Although, weak environmental regulatory institutions often undermine conventional command-and-control policies in these countries [1], various techniques and methods of water purification, such as disinfection, have already been applied. During disinfection, vegetative forms of microorganisms are destroyed or deactivated through interfering with cell activity (e.g., changes in cell permeability, enzyme activity and cell division). It is believed that in the history of humanity, disinfection is one of the most important achievements in the health protection [2]. Improvements in sanitation facilities and water-treatment methods have reduced the spread of many infectious diseases (e.g., cholera and dysentery), whereas the sterilization practices in hospitals have prevented the transition of infectious pathogens to patients. Water disinfection is usually carried out by chlorination, and sometimes by more expensive methods, such as UV irradiation and ozonation. Although, chlorine can inactivate some microorganisms completely, it may also negatively influence water environment, and even animal and human health, e.g., the chlorination by-products are mutagenic and carcinogenic [3]. Moreover, chlorine disinfection has only a low effect on some pathogenic protozoa (e.g., *Cryptosporidium sp*. responsible for diarrhoea and even the death of immunocompromised patients) and viruses (e.g., norovirus). In contrast, UV disinfection is highly efficient against various microorganisms (even viruses) since absorption of UV light (λ = 253.7 nm) by nucleic acids induces the damage of genetic information and inactivation. However, it should be pointed out that chlorine disinfection is often complementary used because: (i) some viruses have low sensitivity to UV irradiation (e.g., adenovirus), and (ii) the lack of residual disinfection of UV irradiation (only during treatment/direct irradiation). Ozone disinfection is also efficient for the degradation of microorganisms, either directly by ozone molecules or by formed oxidative radicals. However, it should be reminded that ozone itself is harmful and can initiate the formation of toxic compounds. Moreover, ozonation and UV irradiation are quite expensive technologies considering both investment and exploration costs.

Accordingly, inorganic antimicrobial agents have been extensively investigated for water purification since they are stable and environmentally safe. Historically, noble metals such as gold, silver and copper have been used since ancient times for the treatment of many diseases and illnesses or for the disinfection of water. In ancient Greece and Rome, the silver or gold dishes left in the sun were believed to protect people against pathogenic microorganisms, and silver coins thrown into the water prolonged its freshness [4–6]. Recent advances in nanotechnology enable to utilize size-dependent properties of nanomaterials, such as high specific surface area, high reactivity, quantum-size effects and plasmonic properties. Nanosilver and nanogold have already been used for various antimicrobial applications, such as (i) ionization techniques, (ii) membrane technologies to reduce biofouling of sanitation devices, (iii) development of sensors with high sensitivity and selectivity for pathogen detection, (iv) everyday products (cosmetics, shoes, clothes and dental treatment). For example, slowly released nanosilver has been used in a variety of domestic appliances, such as washing machines (e.g., Samsung SilverNanoHealth technology). Moreover, antibacterial properties of noble metals have been applied for medical purposes, including healing of burn wounds (bandage and dressing), and protection against infection (braces and catheters).

Photocatalysis is considered as one of the best methods for environmental purification since additional chemical compounds, such as strong oxidants (ozone, hydrogen peroxide or chlorine) [7–14], are not introduced into the environment [15–16]. The energy consumption is also much lower than that of other advanced oxidation technologies (AOTs), e.g., wet air oxidation [17], supercritical water oxidation [18], or H_2_O_2_/UV-C [19]. That is because UV-A lamps or even solar radiation can be used for photocatalyst activation [20]. Titanium(IV) oxide (titania) is the most widely used semiconductor photocatalyst used in treatment of water/wastewater and air [21]. The major barrier for common application of titania photocatalysis is the low activity under solar radiation (mainly due to wide band gap of titania of 3.0–3.2 eV and the recombination of charge carriers). There are various ways to improve photocatalytic performance of titania photocatalysts: (1) to inhibit the recombination of charge carriers, and (2) to extend its working abilities to the visible-light region, for example, by morphology design [22–26], surface modification [27–32], doping [33–36], or the formation of heterojunctions with other semiconductors [37–40]. Modification with noble metals seems the most promising as it is well known that under UV irradiation, noble metals work as an electron pool retarding the recombination of charge carriers [41–43], whereas under visible-light irradiation titania is activated by the plasmonic properties of noble metals (“plasmonic photocatalysis”) [44–45]. Accordingly, it has been proposed that the combination of the antimicrobial properties of noble metals with the high photocatalytic activity of modified titania should result in a high purification efficiency of noble metal-modified titania [6,46–52]. Indeed, in our recent study, noble metal-modified faceted anatase titania (octahedral anatase particles; OAP) have shown high activity in both the decomposition of organic compounds and of microorganisms (*E. coli* and *C. albicans*) [48]. It has been found that both the intrinsic properties of silver and the photocatalytic activity of silver-modified titania are responsible for the high antibacterial activity under visible-light irradiation, whereas the size and shape of silver nanoparticles (NPs) and the aspect ratio of titania affect the antifungal activity [29]. In the present study, antimicrobial properties of commercial titania samples modified with noble metals have been investigated in order to further clarify (1) the mechanism of antimicrobial action of plasmonic photocatalysts, (2) key factors of the high activity against various microorganisms, (3) the correlation between the inactivation and complete decomposition of bacteria, (4) the inhibition of filamentous fungal growth and sporulation by using a new evaluation method.

## Results and Discussion

### Characterization of titania samples modified with NPs of silver and gold

**Figure 1:**
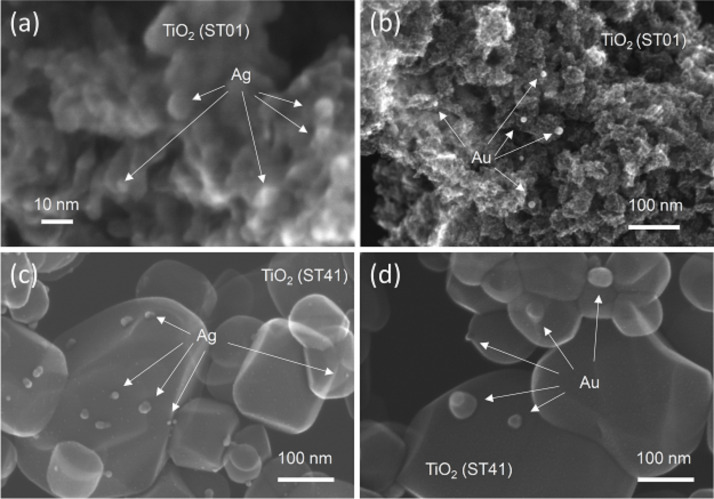
STEM images of modified titania samples: (a) Ag/TiO_2_(ST01), (b) Au/TiO_2_(ST01), (c) Ag/TiO_2_(ST41) and (d) Au/TiO_2_(ST41).

Six commercial titania samples were modified with gold or silver by photodeposition [53–54]. In brief, during photodeposition, metal cations were reduced by photogenerated electrons on irradiated titania. To enable a efficient deposition of metals on titania, photodeposition was carried out in 50 vol % methanol as a hole scavenger and under anaerobic conditions to avoid electron scavenging by oxygen. To find the key factors of antimicrobial activity, various titania samples modified with noble metal NPs (NMNPs) were investigated. The NMNPs differ in crystallographic composition, crystallite sizes and specific surface area. It was confirmed that properties of titania influenced the resultant properties of NMNPs [53], as shown in Table 1. Min et al. proposed that gold clusters were preferentially deposited on surface defects of the support [55]. Therefore, fine titania with a high number of defects (electron traps, ETs) induced the formation of fine and well-dispersed NMNPs on titania surface, as shown in Figure 1 (top). In contrast, titania with large crystals (low number of ETs) induced the aggregation of NMNPs on its well-crystallized surface (Figure 1 (bottom)). It was found that silver formed smaller NPs than gold, as shown in Figure 1 (left and right, respectively).

**Table 1:** Properties of titania photocatalysts used for metal deposition and crystallite sizes of gold and silver NPs.

titania	phase^a^	size^b^/nm	ET^c^/mmol·g^−1^	BET^d^/m^2^·g^−1^	NP size^e^/nm	
					Au	Ag

ST01	anatase	8^f^	84^f^	298^f^	8	0.9^g^
ST41	anatase	205^f^	25^f^	11^f^	29	20
TIO12	anatase	6^f^	111^f^	290^f^	14	—
FP6	anatase/r	12	—	97	—	6
TIO6	rutile	15^f^	242^f^	100^f^	14	—
STF10	rutile/a	102	71^f^	12^f^	23	22

^a^crystal phase (r: small content of rutile and a: small content of anatase), ^b^crystallite size of the dominant titania phase, ^c^content of electron traps, ^d^specific surface area, ^e^crystallite size of metal NPs, ^f,g^data reported previously in [56–57], respectively.

**Figure 2:**
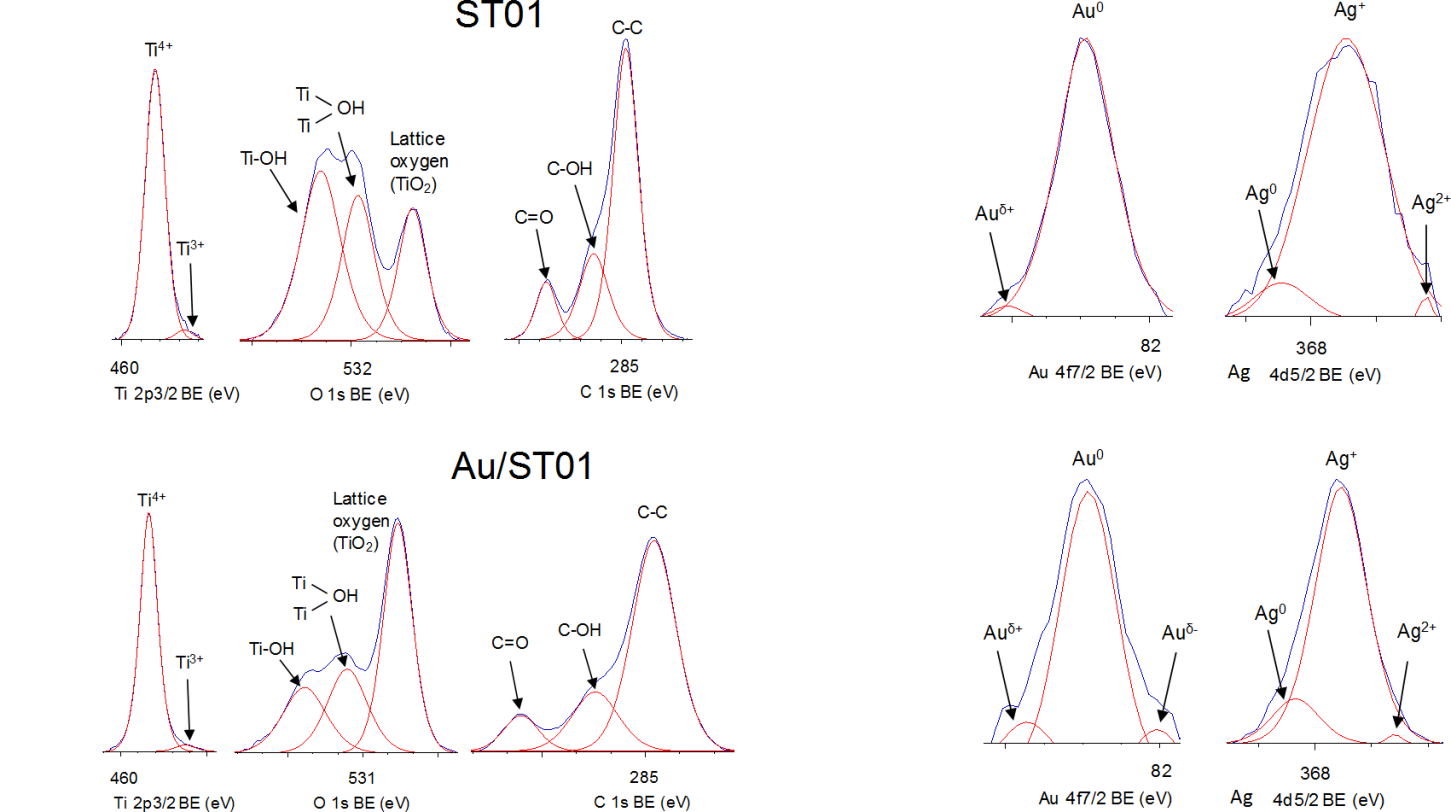
XPS spectra for bare (top left) and gold-modified (bottom left) TiO_2_(ST01) sample, and deconvoluted peaks of Au 4f_7/2_ (center) and Ag 3d_5/2_ (right) for metal-modified ST01 (top) and ST41 (bottom).

**Figure 3:**
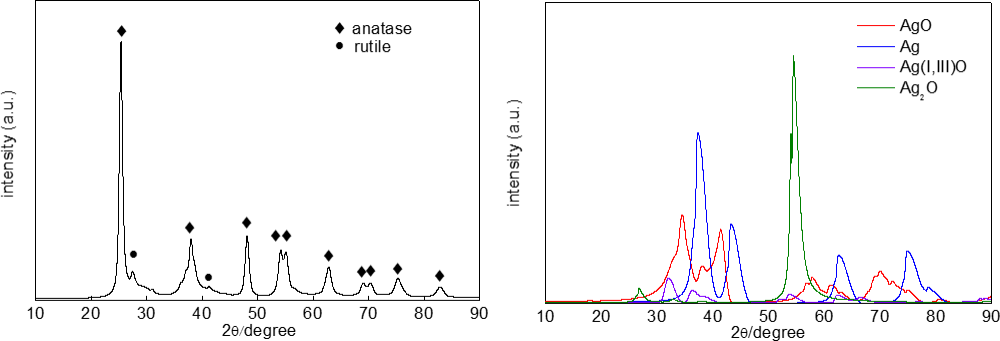
XRD patterns of Ag/TiO_2_(FP6): (left) original pattern, and (right) after subtraction of titania peaks.

To investigate the surface properties of the deposited noble metals, XPS analysis was performed, and exemplary data are shown in Figure 2. XPS peaks of Au 4f_7/2_ and Ag 3d_5/2_ were assigned to two and/or three components, i.e., Au^δ+^, Au^0^ and Au^δ−^ for binding energies (BE) of ca. 83.0, 82.5 and 82.0 eV, respectively, and Ag^0^, Ag^+^ and Ag^2+^ for BE of ca. 368.4, 367.4 and 366.3 eV, respectively. It was found that gold existed mainly in zero valent form, whereas silver existed in both zero valent and oxidized forms (mainly as Ag^+^). It is proposed that although zero valent silver NPs were formed during photodeposition (brown colour of suspension, which is characteristic for spherical silver NPs with localized surface plasmon resonance (LSPR) at ca. 410–430 nm), they were easily oxidized under ambient conditions, and the resultant silver deposits on titania were composed of a zero valent silver core and a silver oxide shell. XRD analysis confirmed XPS data showing silver in three oxidation states (Ag(0), Ag(I) and Ag(II)), as shown in Figure 3.

**Figure 4:**
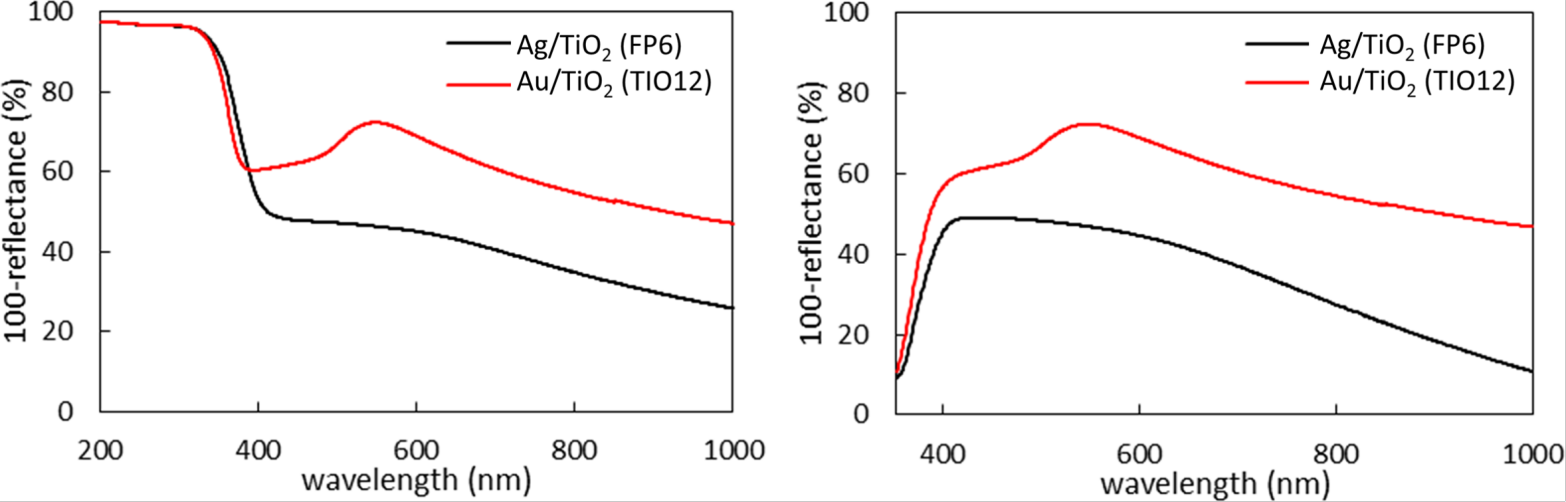
DRS spectra of Ag/TiO_2_(FP6) and Au/TiO_2_(TIO12) taken with BaSO_4_ (left) and respective bare titania (right) as a reference.

All modified titania samples were coloured due to LSPR of NMNPs. Gold-modified samples were violet (light violet for larger titania and gold NPs (Au/TiO_2_(ST41), Au/TiO_2_(FP6)) and dark violet for smaller particle sizes (Au/TiO_2_(TIO12), Au/TiO_2_(ST01)), and silver-modified samples were brown-violet. Exemplary diffuse reflectance spectra (DRS) are shown in Figure 4. It should be pointed out that LSPR of silver could overlap with photoabsorption of titania, and thus to obtain clear LSPR peak of silver, DRS taken with bare titania as reference is recommended (Figure 4 (right)). The intrinsic absorption of anatase titania was observed at wavelengths shorter than 400 nm (*E*_g_ > 3 eV), and LSPR peaks appeared at longer wavelengths for gold NPs (λ_max_ at ca. 560 nm) than for silver NPs (λ_max_ at ca. 450 nm), correlating well with reported data for spherical NPs of gold and silver [58].

### Antibacterial activities of plasmonic photocatalysts

At first, the antibacterial properties of bare titania samples were investigated, and data are presented in Supporting Information File 1 (Table S1). In the dark, antibacterial properties of bare titania samples were very low reaching 15.4%, 16.4% and 23.7% of mortality after 45 min of treatment with TiO_2_(ST01), TiO_2_(ST41) and TiO_2_(TIO6) samples, respectively. Under irradiation with visible light, an increase in antibacterial properties was noticed reaching 23.2%, 25.8% and 27.9%, respectively. Under UV irradiation, significant increase in activity was observed, especially for the fine anatase sample (ST01, 93.1%). Modification with gold caused a slight increase in activity under UV irradiation reaching 96.2%, 94.2% and 77.5%, respectively. An increase in photocatalytic activity after titania modification with NPs of NMs is not surprising, due to a decrease in charge-carrier recombination since noble metals work as an electron sink [54]. The most interesting finding was a significant enhancement of activity under vis irradiation and in the dark. Under vis irradiation the antibacterial activity was increased four times for ST01 and TIO6 samples and three times for ST41 sample, and in the dark it was increased two, three and four times for TIO6, ST01 and ST41, respectively. It is proposed that inactivation of bacteria under visible light irradiation is mainly caused by plasmonic photocatalysis, i.e., the activation of titania by LSPR of gold with a probable electron transfer from gold NPs to the conduction band (CB) of titania and subsequent reduction of oxygen resulting in the formation of reactive oxygen species (ROS). It is also possible that bacteria could be easier adsorbed on positively charged (electron-deficient) gold NPs, and then directly oxidized with simultaneous electron transfer to gold NPs keeping them in the initial zero valent state. A similar mechanism was proposed for decomposition of organic compounds under vis irradiation [53]. The increase in dark activity of titania samples after modification with gold could be caused by an extracellular electron transfer between bacteria and gold, i.e., the surface of gold-modified titania could abstract respiration-active electrons from bacteria, inducing bacterial death [59]. In addition, silver and gold could affect proper transport through the plasma membrane by alteration of the membrane viscosity and disruption of ionic pumps [60]. It is assumed that fine gold NPs more effectively clogged the channels (called porins), which allowed for passive transports of various ions, amino acids and other important molecules. This mechanism is independent on light action [61–63]. In this mechanism, the photocatalyst properties such as particle shape, size and content of NPs could influence the efficiency on interaction between NPs and bacteria [64].

**Figure 5:**
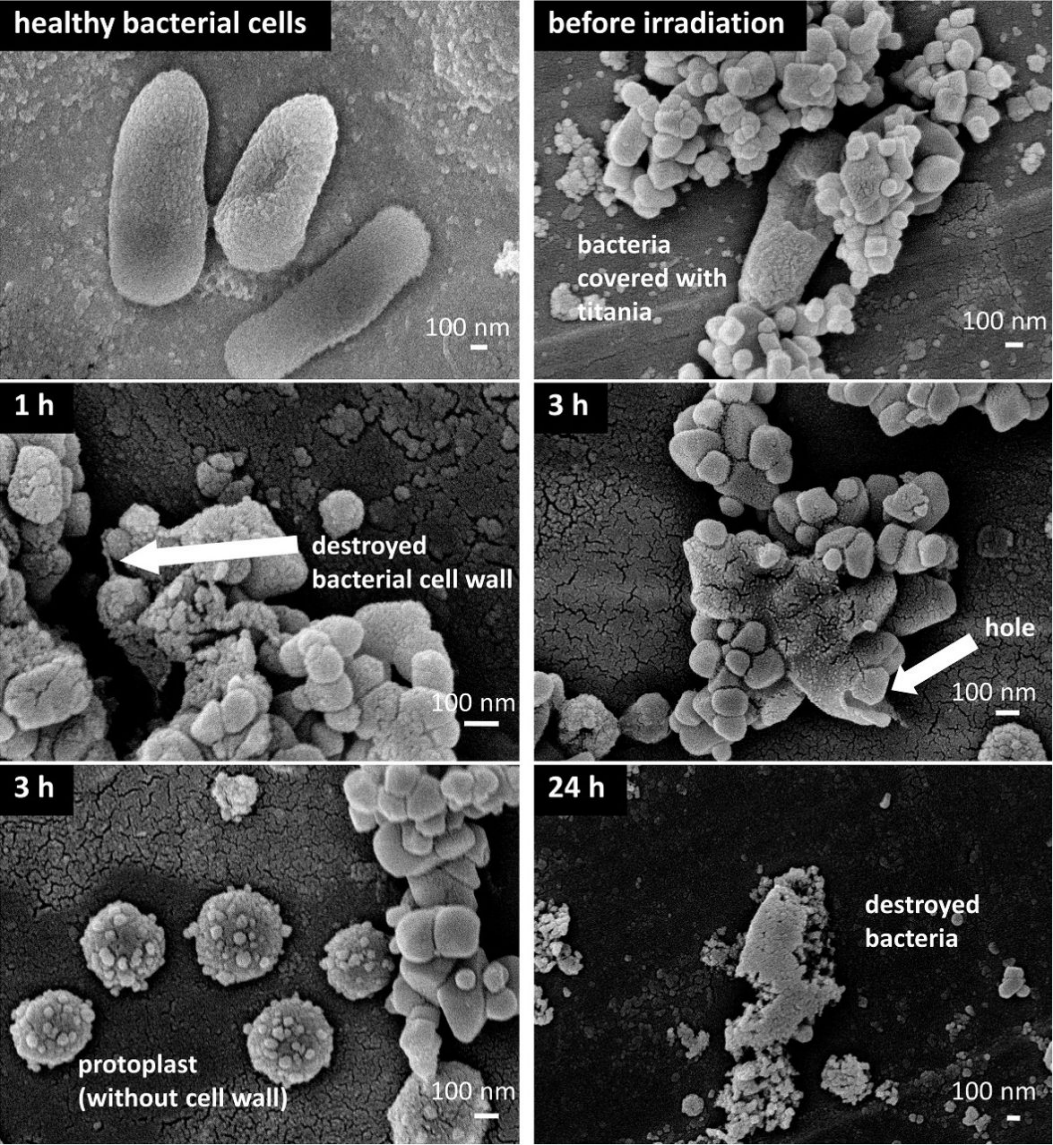
SEM images of the decomposition of bacterial cells under vis (λ > 420 nm) irradiation on Ag/TiO_2_(ST41) photocatalyst.

To evaluate the difference between dark activity and plasmonic photocatalysis, decomposition of bacterial cells in titania suspension with simultaneous measurements of evolved carbon dioxide was studied for bare and modified titania with gold or silver. Our preliminary studies on TiO_2_(ST41) photocatalyst showed that although inactivation of bacterial cells was similar for bare and silver-modified samples (eight orders of decrease after three hours of vis irradiation and one order in the dark), only the silver-modified samples under vis irradiation caused continuous evolution of carbon dioxide suggesting a mineralization of bacterial cells [54]. To check if bacterial cells were really decomposed, scanning electron microscopy (SEM) observations were performed and the obtained data are shown in Figure 5. It was found that 1 h of vis irradiation was sufficient to initiate the destruction of bacterial cell walls, and 3 h of irradiation resulted in the decomposition of bacterial cells with clear formation of protoplast.

**Figure 6:**
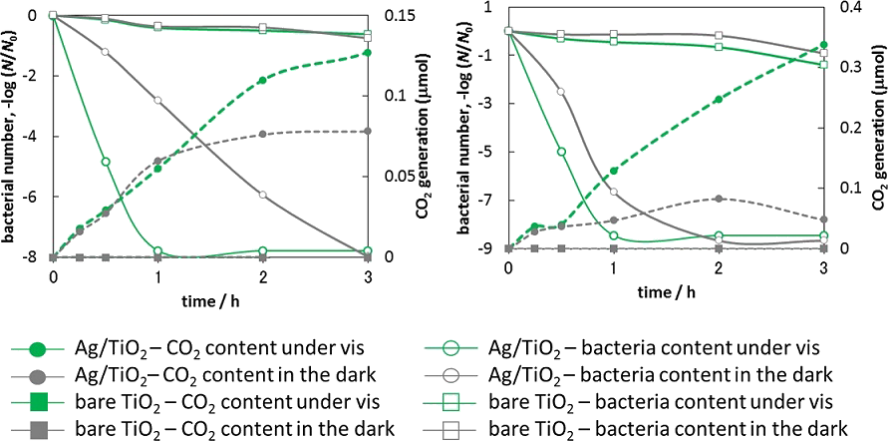
Number of *E. coli* bacteria (closed symbols) and evolution of CO_2_ (open symbols) during inactivation of bacterial cells in the dark (grey symbols) and under vis irradiation (λ > 420 nm; green symbols) on bare (squares) and modified titania (circles): (left) TiO_2_(ST01) and Ag/TiO_2_(ST01), (right) TiO_2_(FP6) and Ag/TiO_2_(FP6).

For the other silver-modified samples (Ag/TiO_2_(FP6) and Ag/TiO_2_(ST01)) a similar tendency was observed, i.e., continuous evolution of carbon dioxide was observed for silver-modified samples under vis irradiation as shown in Figure 6. Carbon dioxide also evolved on silver-modified samples in the dark, but only during the first two hours. In contrast to Ag/TiO_2_(ST41), for which similar inactivation of bacteria was observed in the dark and under vis irradiation [35], a significantly larger activity for silver-modified samples was noticed under vis irradiation than that in the dark. The higher activity of Ag/TiO_2_(FP6) compared to Ag/TiO_2_(ST01) in the dark is presumably caused by larger NPs of silver in Ag/TiO_2_(FP6) (Table 1), because there is a tendency that antibacterial activity increases with an increase in the size of silver NPs [65]. Indeed, the highest activity in the dark was obtained for the large NPs of silver in the Ag/TiO_2_(ST41) sample. There are two possible explanations for the larger activity under vis irradiation: (1) bacteria inactivation by plasmonic photocatalysis (generation of ROS under vis irradiation) or (2) formation of larger amounts of positively charged silver and its subsequent release into bacteria–titania suspension (also through a plasmonic mechanism, as discussed above for gold). In order to clarify the exact mechanism under vis irradiation, the dependence of activity on the irradiation wavelength should be scrutinized, which is presently done. At the moment, it is clear that the activity of bare titania in the dark and under vis irradiation is negligible compared to the high activity of the silver-modified samples. For comparison, a gold-modified sample (Au/TiO_2_(TIO12)) was also tested, and the obtained data are shown in Supporting Information File 1 (Figure S1). For bare titania (TIO12, fine anatase), similar to other titania samples (ST01 and FP6 (Figure 6)), negligible activities under vis irradiation and in the dark, were noticed. A negligible activity of the gold-modified sample under dark conditions was observed, which was even lower than that of bare titania. It is proposed that very fine gold NPs on titania could disturb the binding of titania to the bacterial cells as a result of active site blocking by gold [66]. Thus a lower antibacterial activity was obtained. Under vis irradiation, a high antibacterial activity was observed, but significantly lower than that of the silver-modified samples (two to four orders of magnitude). Although, the activity of the gold-modified sample was lower, it is clear that it originated from plasmonic activation of titania. Probably, this is the first evidence proving antibacterial activity of plasmonic photocatalysts under visible-light irradiation (λ > 420 nm), resulting only from plasmonic activation of titania. There was no activity in the dark and an inactivity of bare titania. Plasmonic activity of other photocatalysts has been reported, but silver-modified samples, also exhibit activity in the dark due to the antimicrobial effect of silver [26,48,67–68]. Although, gold-modified titania exhibited antibacterial activity under visible-light irradiation, the bacteria killing rate was much slower than that of the silver-modified samples (due to the high silver activity in the dark). Accordingly, it is proposed that silver-modified samples are more promising as antibacterial agents, due to high efficiency (both in the dark and under vis irradiation) as well as the lower price of silver.

### Antifungal activity of plasmonic photocatalysts

High humidity, positive temperature and lack of proper ventilation are significant factors affecting the growth of mould fungi in indoor environment. Some moulds produce spores that pose a serious threat to human health, e.g., chronic fatigue and allergic asthma. Long-term exposure to indoor moulds aggravates mentioned symptoms and can promote new ones. Moreover, fungi as more complex microorganisms than bacteria and viruses are much more resistant to antimicrobial agents. Therefore, there is a great need to develop new efficient antifungal materials. Regarding this, the antifungal properties of plasmonic photocatalysts have been tested. At first, experiments were performed by disc diffusion, as exemplary shown in Supporting Information File 1 (Figures S2–S5). Small (0–5 mm) inhibition zones were observed only for *Candida albicans* for paper discs impregnated with Ag/TiO_2_(ST41) under vis irradiation. Consequently, it can be assumed that silver and gold ions are not releases from modified titania to the medium. All paper discs impregnated with silver-modified titania (Ag/TiO_2_(ST41) and Ag/TiO_2_(ST01)) and gold-modified titania (Au/TiO_2_(ST41) and Au/TiO_2_(ST01)) were avoided by all tested mould fungi under vis irradiation. This suggests that the antifungal effect is mainly associated with photocatalytic action. This effect was not observed for bare titania samples.

Mycelia of mould fungi, e.g., *Aspergillus melleus*, displayed a colour change in direct contact with paper discs impregnated with Ag/TiO_2_(ST01) and Ag/TiO_2_(ST41) in the dark and under vis irradiation due to inhibited sporulation. That effect was not observed for paper discs impregnated with bare and gold-modified titania. The antifungal study based on disc diffusion showed ambiguous results for silver- and gold-modified titania samples. It may be concluded that a concentration of 1 g/L of Ag- and Au-modified titania is not sufficient to observe a biocidal effect, regardless of the experimental conditions. As it was shown in our previous studies [69–70], the photocatalytic degradation of fungi was much lower than that of bacteria, because of differences in inactivation mechanism and cell-wall structure. The potent activity of Ag-modified titania against yeast (*C. albicans*) was caused by its different morphology and taxonomy group. It is well known that yeast are unicellular fungi, and do not produce mycelia and spores, which makes them the most vulnerable object, i.e., a few times more sensitive towards antifungal agents than other fungi. Auyeung et al. found that only metallic and ZnO NPs showed a potent antimould activity against *A. niger* and *P. chrysogenum* [71].

**Figure 7:**
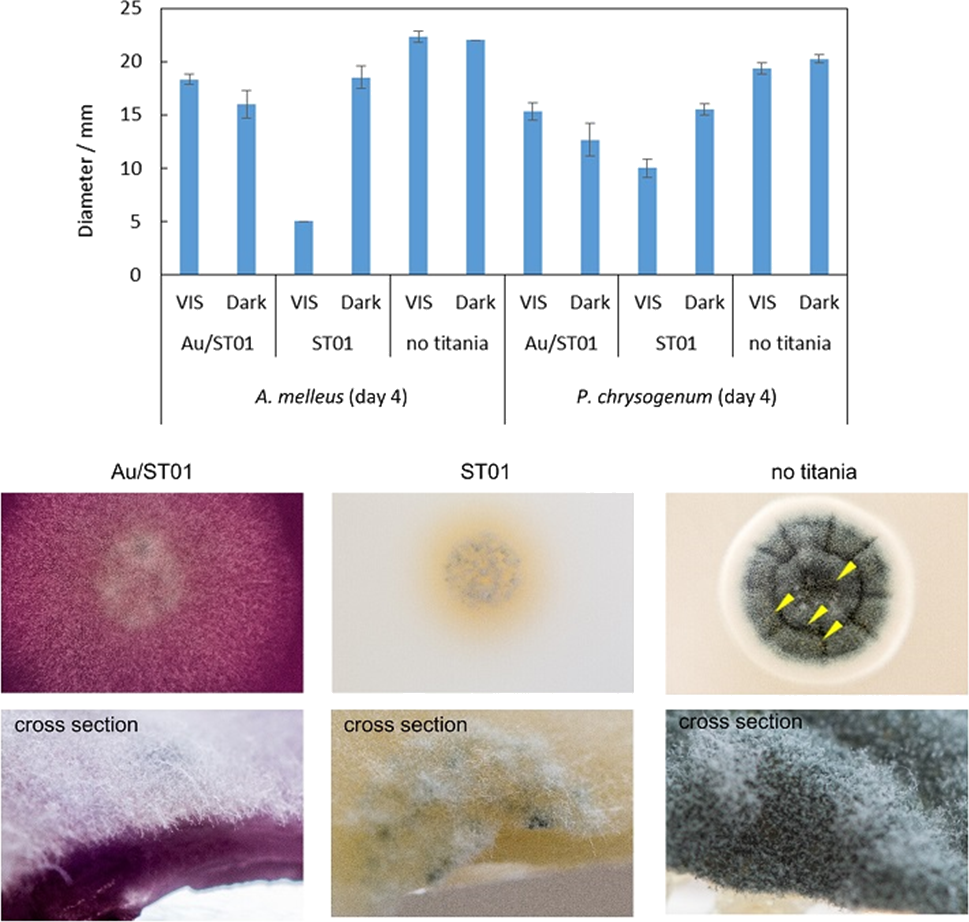
(top) Antifungal properties of bare and gold-modified titania ST01 by a comparison of diameters of colonies after four days of growth (*n* = 6, mean ± S. D.), and (bottom) representative photograph of *P. chrysogenum* cultivated for four days under fluorescent-light irradiation. Arrowheads indicate the droplets.

Despite the low antifungal activity of silver-modified titania, the inhibition zones around paper disks may be considered as positive results indicating a high stability of photocatalysts. However, it should be concluded that the antifungal properties of silver-modified titania are not attractive for commercial application. Moreover, the small inhibition zones around discs were difficult to evaluate quantitatively. Therefore, the “poisoned food” method (fungal growth on titania/MEA support) with gold-modified titania was used for further investigations. At first, bare titania samples were examined, and obtained data are shown in Supporting Information File 1 (Figure S6). It was found that fungi, especially *A. melleus*, were sensitive to photocatalysis, and the highest activity was obtained for fine titania samples especially under vis irradiation. Interestingly, for *P. chrysogenum* and *A. niger* the same activity of bare titania FP6 was observed in the dark and under irradiation. It should be mentioned that both titania samples (ST01 and FP6) are highly active photocatalysts for various photocatalytic reactions, e.g., oxidative decomposition of acetic acid and dehydrogenation of methanol [72]. FP6 has larger crystals (smaller specific surface area), and thus its lower activity under irradiation is reasonable. Not only the diameter of the mycelium, but also the level of sporulation should be estimated for antifungal activity tests, and summarized data for mycelium growth and sporulation are shown in Supporting Information File 1 (Table S2**,** and exemplary photographs of fungal growth in Figure S7). It was confirmed that titania ST01 was more efficient than titania FP6. Next, gold-modified samples were examined, and antifungal properties of gold were confirmed in the dark, as shown in Figure 7 and in Supporting Information File 1 (Table S3).

Surprisingly, under fluorescent irradiation bare titania was more active than gold-modified titania. Noble metal-modified titania samples are usually more active under both UV and visible light irradiation than bare titania samples (see studies for sixteen commercial titania samples modified with gold [53] and silver [43,54]). However, in the near-UV range and in some cases, bare titania shows larger photocatalytic activity [45]. It should be reminded that the titania samples here are commercial samples, and thus with small content of impurities and defects, which can increase the visible-light activity of titania by narrowing of the band gap. The adsorbed gold on the titania surface would disturb this intrinsic vis response at 390–420 nm. Moreover, it should be clarified that the lower inhibition of mycelium growth does not unequivocally mean a lower antifungal action, since sporulation and possible mycotoxin generation must be also considered. Therefore, a decrease in sporulation and the inhibition of droplet formation (with possible presence of mycotoxins) on gold-modified titania indicate that gold-modified titania could be used as efficient antifungal agent.

**Figure 8:**
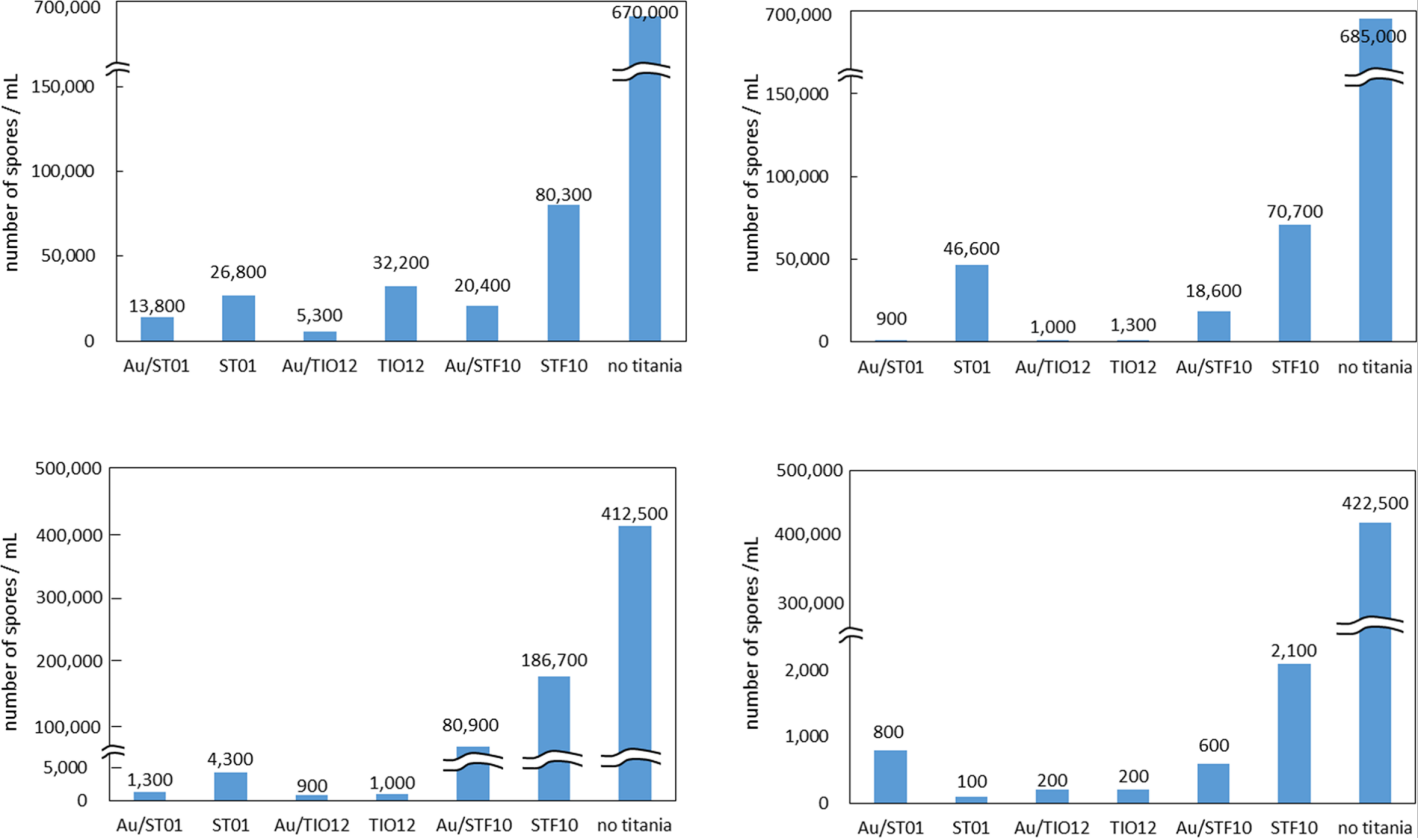
Sporulation after five days of growth of *P. chrysogenum* (top) and *A. melleus* (bottom) under vis light (left) and in the dark (right).

Antifungal properties were also confirmed for other gold-modified titania samples (TIO12 and STF10), as shown in Supporting Information File 1 (Figures S8–S9). Although, the antifungal effect was clear, the evaluation of the overall antifungal activity was quite troublesome, due to the difficult differentiation between the colours of mycelium and support (modified-titania). Therefore, a new method, namely “spore counting” was applied (Supporting Information File 1, Figure S10), and the obtained data are shown in Figure 8. In the literature, contradictory opinions on the influence of light on fungi have been presented. Light can stimulate or inhibit fungal growth or be without any effect [73]. Most filamentous fungi are photophobic [74]. Light could limit (or even completely inhibit) germination, probably due to an increase in temperature during irradiation [75]. The inhibition effect increases with an increase in light energy, i.e., UVB (280–320 nm) is more harmful than UVA (320–400 nm). UV light in the solar spectrum is considered to be responsible for inhibition of mycotoxin generation [75]. It is known that fungi are heterotrophic organisms, and thus light is not necessary for their growth. However, it has been also reported that light may act on enzyme levels and metabolite secretion in fungal cells [75]. Our results clearly demonstrate that irradiation does not influence the sporulation of *A. melleus* and *P. chrysogenum* in the absence of titania. However, a significant effect on sporulation was observed for bare titania. Moreover, gold slightly enhanced this effect depending on the fungi species. *P. chrysogenum* was sensitive to light, which stimulated its growth, whereas irradiation almost did not influence the groth of *A. melleus*.

To the best of our knowledge, this is the first report on the effect of gold-modified titania on sporulation of mould fungi. It is clear that although a negligible inhibition of mycelium growth was observed (Figure 7, and Figure S8 and Figure S9 and Table S3 in Supporting Information File 1), sporulation was inhibited almost completely (99.8%) on slants supplemented with Au/TiO_2_. In order to understand the function of Au NPs in the inhibition of sporulation, colloidal Au NPs (ca. 12 nm) were investigated by the same method (Figure S11, Supporting Information File 1). It was found that unsupported Au NPs did not show inhibition of sporulation in the dark, and only a slight inhibition under visible-light irradiation for *A. melleus.* Accordingly, it is proposed that Au NPs do not intrinsically inhibit sporulation. It may be concluded that the Au–TiO_2_ heterojunction is necessary to influence the formation of sporangia and/or spores during the growth of mycelium. Although, further investigation is necessary for the mechanism clarification, it is proposed that titania could change the availability of nutrients from media. Suberkropp has already reported that the nutrient availability and allocation of resources are main factors determining fungal reproduction (sporulation) [76]. It is proposed that the main reason of a decrease in media nourishment for mould fungi is probably caused by sorption of macromolecules (e.g., proteins and sugars) on titania, as suggested by Raffaini and Ganzoli [77] and Ellingsen [78].

On the other hand, our previous study showed that Au-modified faceted titania was inactive against *C. albicans*, in contrast to the Ag-modified sample [29]. It is assumed that different results could originate from different structures of the fungi. *C. albicans* is a dimorphic fungus since it grows as yeast, and it neither forms a mycelium nor produces reproductive cells (spores). It is proposed that Ag could penetrate the cell envelope easily and cause significant changes in survival rate, due to the simple cell structure of *C. albicans*. Accordingly, it is proposed that the bactericidal mechanism could be similar to the anti-“candida” mechanism, and different from antifungal mechanism for mould fungi.

Rogawansamy et al. have proposed that the best antifungal agent should inhibit the growth of various fungal genera, whereas less active antifungal agents could only inhibit sporulation or the growth of a limited range of fungi [79]. However, it should be reminded that spores are essential for fungal reproduction by expanding into the environment, and thus causing various allergies and respiratory diseases. Therefore, it is proposed that Au/TiO_2_, which exhibits effective inhibition of sporulation, can be a promising material for reducing health-threatening factors in indoor environment.

## Conclusion

Noble metal-modified titania photocatalysts exhibit good antimicrobial properties in the dark and under visible-light irradiation due to antimicrobial properties of noble metals and plasmonic photocatalysis. The surface properties of photocatalysts are crucial for the antimicrobial effect, and generally, a decrease in particle sizes results in an activity increase, due to a larger interface between microorganisms and photocatalysts. Silver-modified samples show high antibacterial activity (complete decomposition of bacterial cells), whereas gold-modified samples are very active against fungi, especially in the inhibition of sporulation. Therefore, it is expected that bimetallic (Au/Ag) titania photocatalysts should exhibit broad antimicrobial properties. The lack of inhibition zones around disks supplemented with photocatalysts confirms the high stability of photocatalysts and their commercial applicability.

## Experimental

### Preparation of silver- and gold-modified TiO_2_

2 wt % of silver and gold were deposited on six commercial titania samples: ST01 (Ishihara, ST-01), ST41 (Ishihara, ST-41), TIO12 (TAYCA, JRC-TIO-12), FP6 (Showa Denko Ceramics, FP-6), TIO6 (Catalysis Society of Japan, JRC-TIO-6) and STF10 (Showa Denko Ceramics, ST-F10). In brief, TiO_2_ samples were suspended in a 50 vol % aqueous solution of methanol, to which either AgNO_3_ aqueous solution or HAuCl_4_·H_2_O aqueous solution was added, suspensions were purged with argon for 15 min, and irradiated with a 400 W mercury lamp in a thermostatic water bath at 298 K under stirring (500 rpm). The obtained noble metal-modified TiO_2_ samples were centrifuged, washed twice with methanol and twice with Milli-Q water and dried at 378 K.

### Antibacterial activity test

50 mg of the sample was dispersed in 7.0 mL of *E. coli* K12 (ATCC29425) suspension at a concentration of ca. 0.180 Abs at 630 nm (ca. 1–5 × 10^8^ cells/mL) in a test tube with stirring bar and irradiated with a xenon lamp (with CM1 and Y-45 filter; λ > 420 nm) or kept in the dark. Serial dilutions (10^−1^ to 10^−6^) were prepared and aliquots of suspensions were inoculated on Plate Count Agar (Becton, Dickinson and Company, USA) media after 0, 0.5, 1, 2 and 3 h. Media were incubated at 37 °C overnight and then colonies were counted. Simultaneously, generated CO_2_ was measured by FID-gas chromatography.

For SEM observation, 0.1 mL of the mixture of *E. coli* K12 and sample before and after reaction was collected, diluted 10 times with sterile Milli-Q water and mounted on a piece of copper tape. The bacterial cells were fixed by 2.0% glutaraldehyde and 2.0% paraformaldehyde in 30 mM HEPES buffer for 1 h. After fixation, cells were washed with 30 mM HEPES buffer for 1 min, dehydrated by a graded series of ethanol (30, 50, 70, 90, 95 and 99.5% (v/v)) for 10 min each. Then, samples were washed with *tert*-butyl alcohol three times, soaked in it and dried under vacuum. Samples were sputtered with platinum for 60 s from three directions and observed by SEM (JSM-7400F, JEOL).

### Antifungal activity test

The antifungal tests were performed with the following microorganisms: the yeast *Candida albicans*, isolated from patients (throat smear) with immunodeficiency disorders that cause candidiasis (IIT&EE ZUT collection), and mould fungi *Aspergillus niger*, *Aspergillus melleus* and *Penicillium chrysogenum*, isolated from damp basement air (IIT&EE ZUT collection). The antimicrobial activity of the photocatalysts was tested by using disc diffusion [80]. Culture plates were prepared with 20 mL of two solid media: Sabouraud Glucose Agar SGA (BIOCORP, Poland) for *C. albicans* and Malt Extract Agar (Merck, Germany) for mould fungi. Sterilized culture media were poured in Petri dishes and, after solidification of the medium, 0.25 mL of fungal suspension was spread on the plate using an L-rod. The concentration of microorganisms was ca. 10^6^ CFU/mL. The sterile paper discs (Whatman No.1, diameter 5 mm) impregnated with photocatalyst suspension (1 g/L 10 µL/disc) were placed at different locations on the culture plates. The control discs were impregnated with saline solution (0.9% NaCl). One part of the plates was incubated in the dark, while another part was exposed to the indoor light. The natural indoor light from windows (intensity of ca. 120 lx) was strengthened by 8 h of exposure to a halogen lamp (intensity of ca. 400–500 lx). The distance from three halogen lamps to the plates was ca. 50 cm. The temperature was maintained at ca. 30 °C for *C. albicans* and 25 °C for *A. niger*, *A. melleus* and *P. chrysogenum*. The incubation periods were 48 h for *C. albicans* and 72 h for mould fungi. After incubation, the zones of inhibition around the paper discs were investigated.

### Colony growth test

20 g/L of the sample was dispersed in Malt Extract Agar (MEA) medium, sonicated for 1 min, autoclaved at 121 °C for 10 min and poured in Petri dishes. The spores of *A. niger*, *A. melleus* and *P. chrysogenum* were collected with 8.5 g/L NaCl aqueous solution, and the suspension was inoculated on the Petri dishes. Dishes were irradiated with fluorescence light or kept in dark. After three and eight days of exposure the diameters of colonies were measured and the appearances were checked visually.

### Sporulation test

20 g/L of the sample was dispersed in MEA medium, sonicated for 1 min, autoclaved at 121 °C for 10 min and dispensed to the slants. The spores of *A. melleus* and *P. chrysogenum* were collected with 8.5 g/L NaCl aqueous solution, the number of spores were counted by Thoma hemacytometers and 10,000 spores/mL of the suspension was inoculated on the slants. The slants were irradiated with fluorescence light or kept in dark. Triton X-100 (0.05%) in 8.5 g/L NaCl aqueous solution was added to the culture after five days and mixed with a vortex mixer. Collected spores were diluted 10–50 times with 8.5 g/L NaCl aqueous solution, and then counted.

## Supporting Information

File 1 Additional experimental data.
