# Supplementary material for: Noble metal-modified titania with visible-light activity for the decomposition of microorganisms
Source: Beilstein J Nanotechnol. 2018 Mar 7;9:829–41. doi: 10.3762/bjnano.9.77 (PMC5852454; doi:10.3762/bjnano.9.77)
Supplement: File 1 — Additional experimental data. [file Beilstein_J_Nanotechnol-09-829-s001.pdf]

Supporting Information

for

Noble metal-modified titania with visible-light activity for the decomposition of microorganisms

Maya Endo[1], Zhishun Wei[1,2], Kunlei Wang[1,3], Baris Karabiyik[1], Kenta Yoshiiri[1,3], Paulina Rokicka[1,4], Bunsho Ohtani[1,3], Agata Markowska-Szczupak*[1,4] and Ewa Kowalska*[1,3]

Address: [1]Institute for Catalysis, Hokkaido University, N21 W10, 001-0021 Sapporo, Japan, [2]School of Materials and Chemical Engineering, Hubei University of Technology, 430068 Wuhan, China, [3]Graduate School of Environmental Science, N10 W5, 060-0810 Sapporo, Japan and [4] Institute of Inorganic Technology and Environment Engineering, West Pomeranian University of Technology, Szczecin, Pulaskiego 10, 70-322 Szczecin, Poland

Email: Ewa Kowalska* - kowalska@cat.hokudai.ac.jp;
Agata Markowska-Szczupak* - agata@erb.pl

* Corresponding author

Table S1: The mortality rate (%) of *E. coli* bacteria

	TiO_2(ST01)	TiO_2(TIO6)	TiO_2(ST41)	Au/TiO_2(ST01)	Au/TiO_2(TIO6)	Au/TiO_2(ST41)
Dark	15.38	23.72	16.40	50.00	43.85	63.22
Vis	23.20	25.75	27.92	97.10	94.64	80.41
UV-A	93.12	64.74	63.23	96.23	77.47	94.23

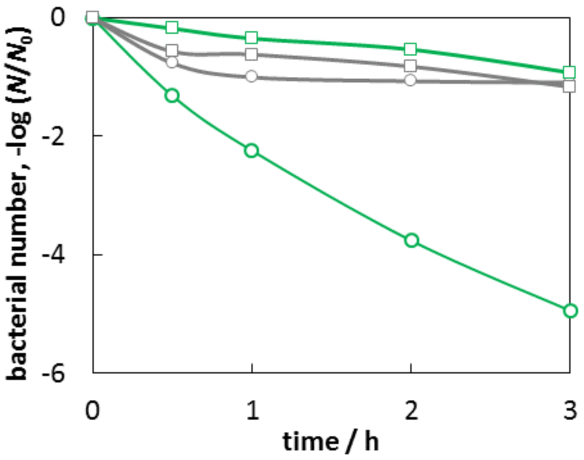

Figure S1: Number of *E. coli* bacteria during inactivation of bacterial cells in the dark (grey symbols) and under vis irradiation ($\lambda > 420$ nm; green symbols) on bare (squares) and gold-modified titania (TIO12, circles).

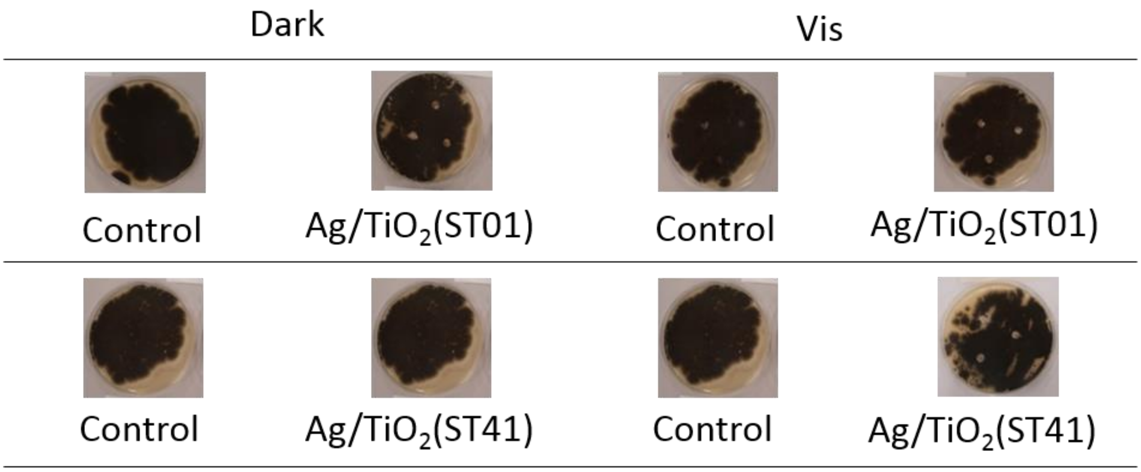

Figure S2: Antifungal activity of silver-modified photocatalyst against *A. niger* after 72 h in the dark condition and under visible light.

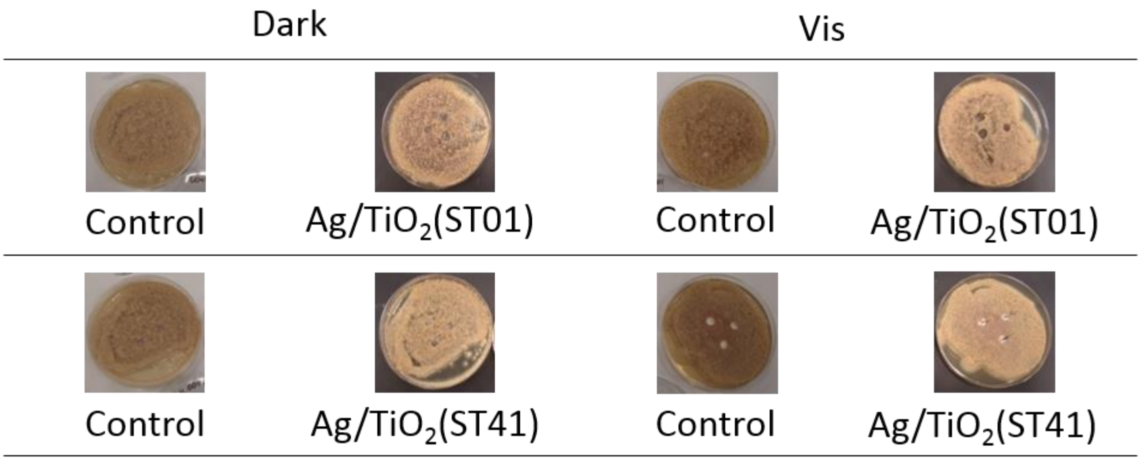

Figure S3: Antifungal activity of silver-modified photocatalyst against *A. melleus* after 72 h in the dark condition and under visible light.

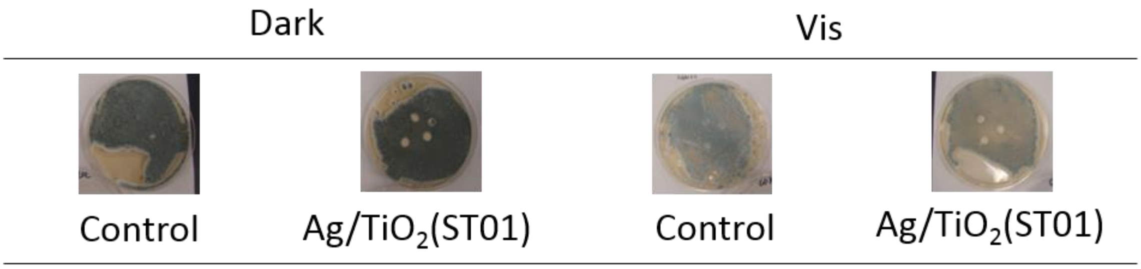

Figure S4: Antifungal activity of silver-modified photocatalyst against *P. chrysogenum* after 48 h in the dark condition and under visible light.

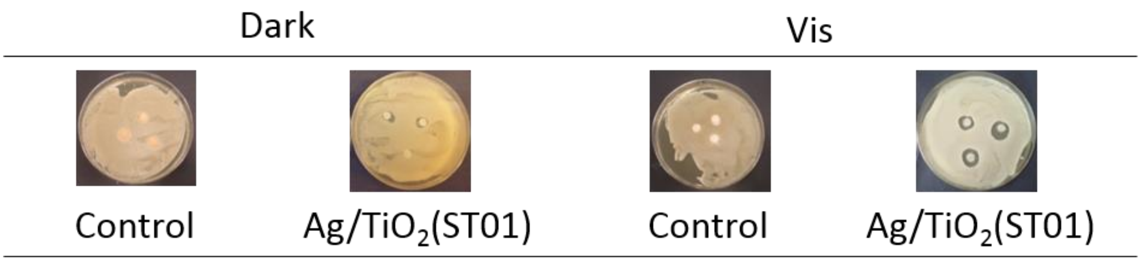

Figure S5: Antifungal activity of silver-modified photocatalysts against *C. albicans* after 48 h in the dark condition and under visible light.

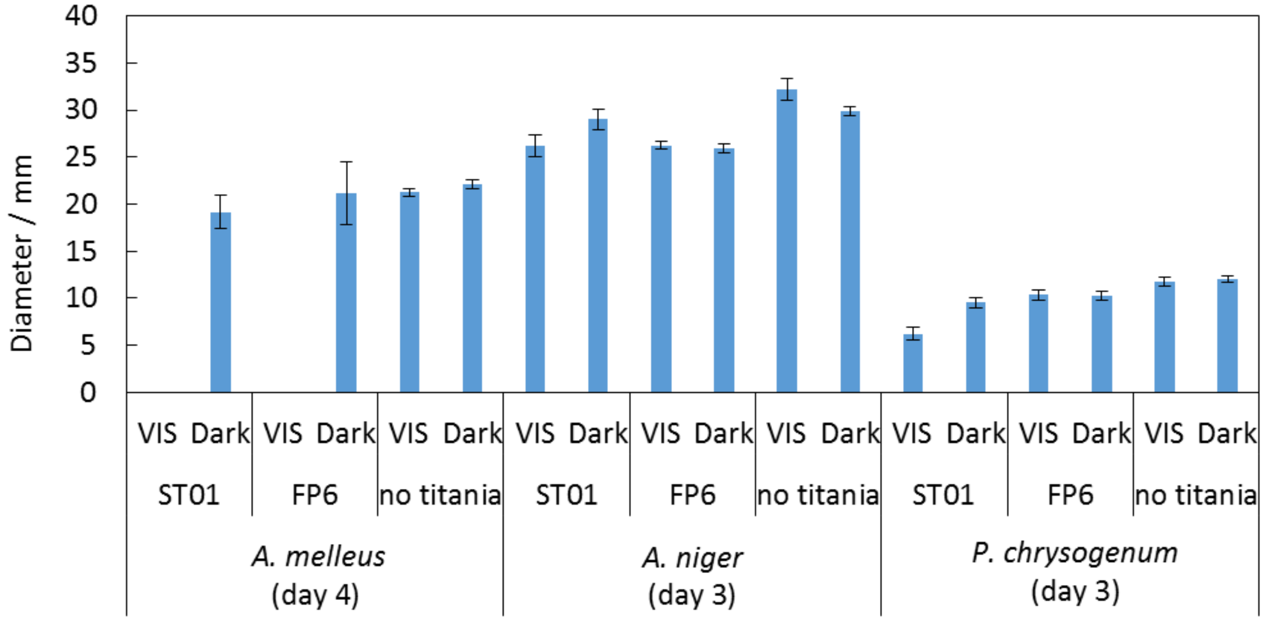

Figure S6: Antifungal activity of bare titania photocatalysts against *A. melleus* (day 4), *A. niger* (day 3) and *P. chrysogenum* (day 3).

Table S2: Summary of antifungal properties of bare titania photocatalysts

			average of mycelium diameter (mm)	S.D.	Student's t test against V/D MEA	spore color	spore size
A. melleus (day 4)	ST01	VIS	0	0	0	-	-
		Dark	19.2	1.72	0.0163	white	-
	FP6	VIS	0	0	0	-	-
		Dark	21.2	3.37	0.5670	brown	middle
	no titania	VIS	21.3	0.42		brown	large
		Dark	22.1	0.49		brown	large
A. niger (day 3)	ST01	VIS	26.2	1.17	0.0011	black	small
		Dark	29.0	1.10	0.1589	white	-
	FP6	VIS	26.3	0.42	0.0001	black	middle
		Dark	25.9	0.49	0.0000	black	middle
	no titania	VIS	32.2	1.17		black	large
		Dark	29.9	0.49		black	large
P. chrysogenum (day 3)	ST01	VIS	6.2	0.68	0.0000	White-blue	ex-small
		Dark	9.5	0.55	0.0002	-	-
	FP6	VIS	10.3	0.52	0.0075	blue	small-middle
		Dark	10.3	0.42	0.0002	blue	small-middle
	no titania	VIS	11.8	0.42		blue	large
		Dark	12.0	0.32		blue	large

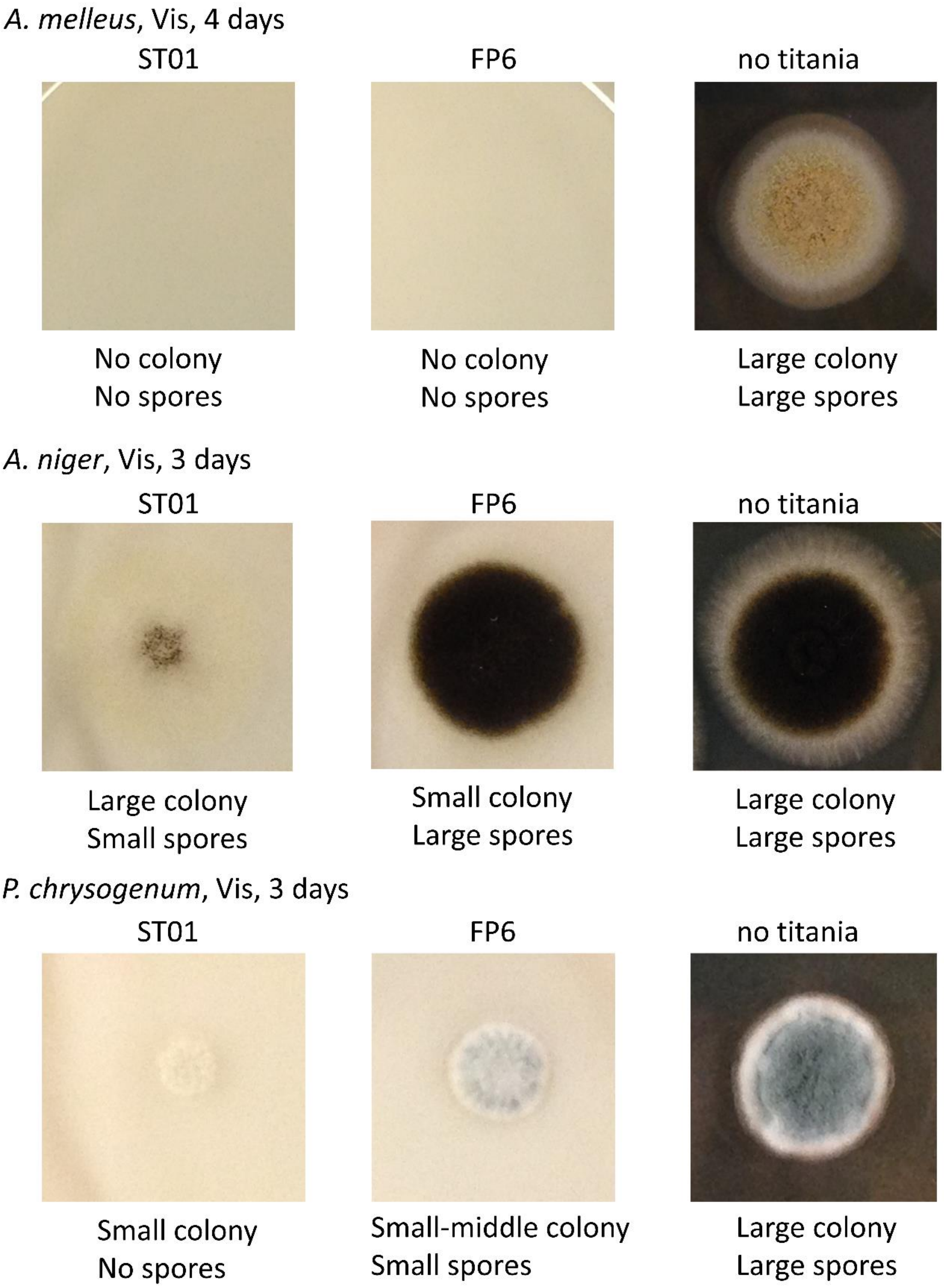

Figure S7: Photographs of mycelium during tests of antifungal properties of bare titania photocatalysts.

Table S3: Summary of antifungal properties of bare and gold-modified titania ST01

			average of mycelium diameter (mm)	S.D.	Student's t test against V/D MEA	spore color	spore size	droplet (toxin)
A. melleus (day 4)	Au/ST01	VIS	18.3	0.52	0.0001	white	ex-small	none
		Dark	16.0	1.26	0.0001	white	ex-small	none
	ST01	VIS	5.0	0.00	0.0000	white	-	none
		Dark	18.5	1.05	0.0004	white	-	none
	no titania	VIS	22.3	0.52		yellow	large	none
		Dark	22.0	0.00		yellow	large	none
P. chrysogenum (day 4)	Au/ST01	VIS	15.3	0.82	0.0000	white	-	none
		Dark	12.7	1.51	0.0001	white	-	none
	ST01	VIS	10.0	0.89	0.0000	blue	middle	none
		Dark	15.5	0.55	0.0000	White-blue	small	none
	no titania	VIS	19.3	0.52		blue	large	yellow
		Dark	20.3	0.42		blue	large	yellow

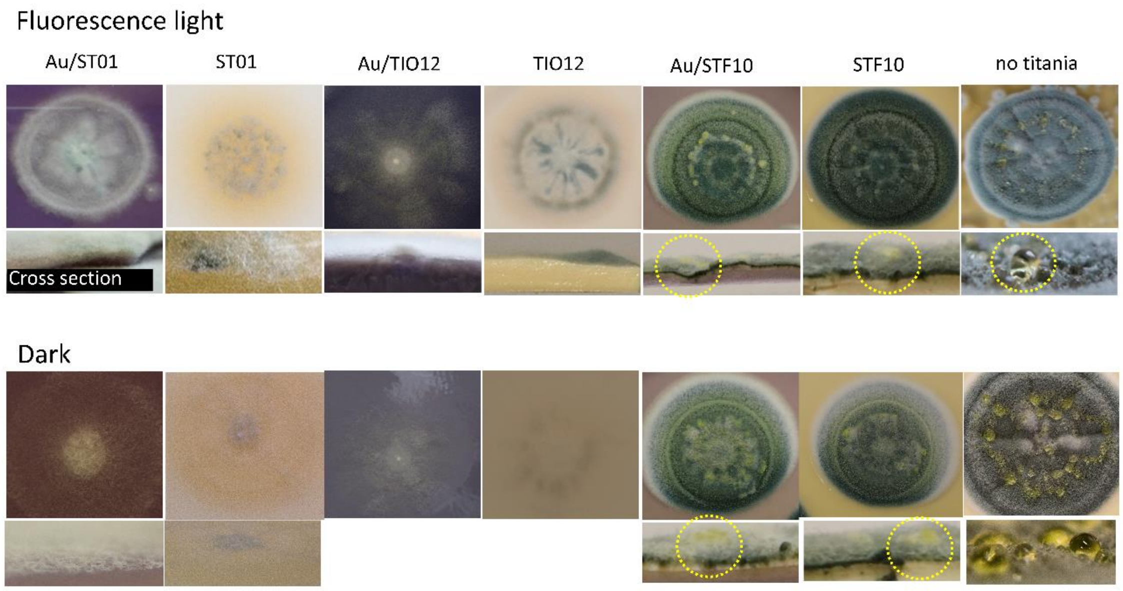

Figure S8: Representative photographs of *P. chrysogenum* with Au/TiO_2(ST01), TiO_2(ST01), Au/TiO_2(TIO12), TiO_2(TIO12), Au/TiO_2(STF10), TiO_2(STF10) and without titania under fluorescence light irradiation and in the dark. Dashed circles indicate the droplets (with possible presence of mycotoxins).

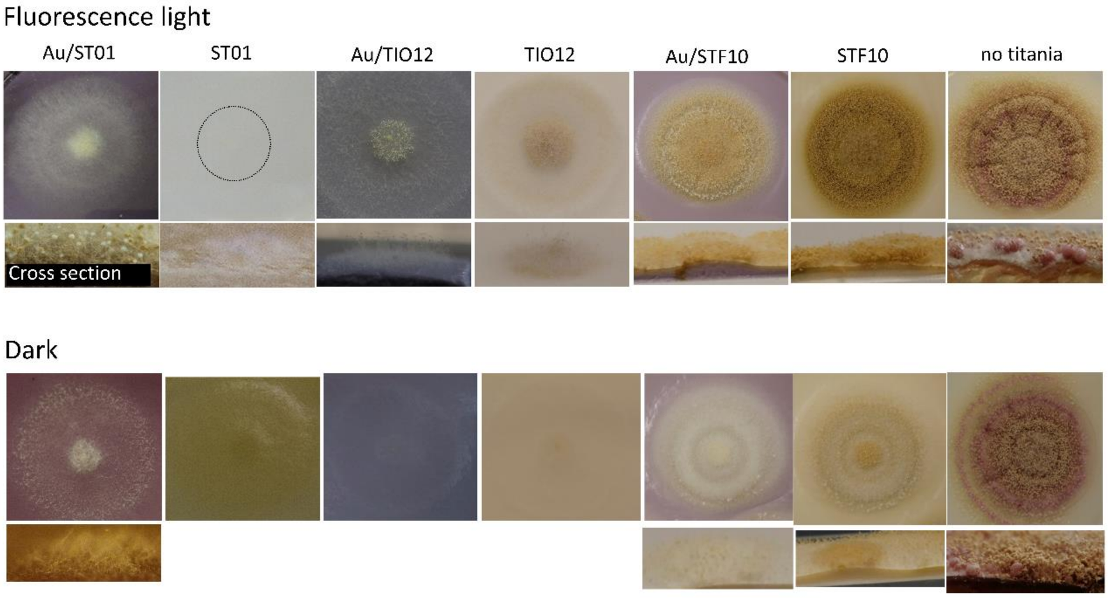

Figure S9: Representative photographs of *A. melleus* with Au/TiO_2(ST01), TiO_2(ST01), Au/TiO_2(TIO12), TiO_2(TIO12), Au/TiO_2(STF10), TiO_2(STF10) and without titania under fluorescence light irradiation and in the dark.

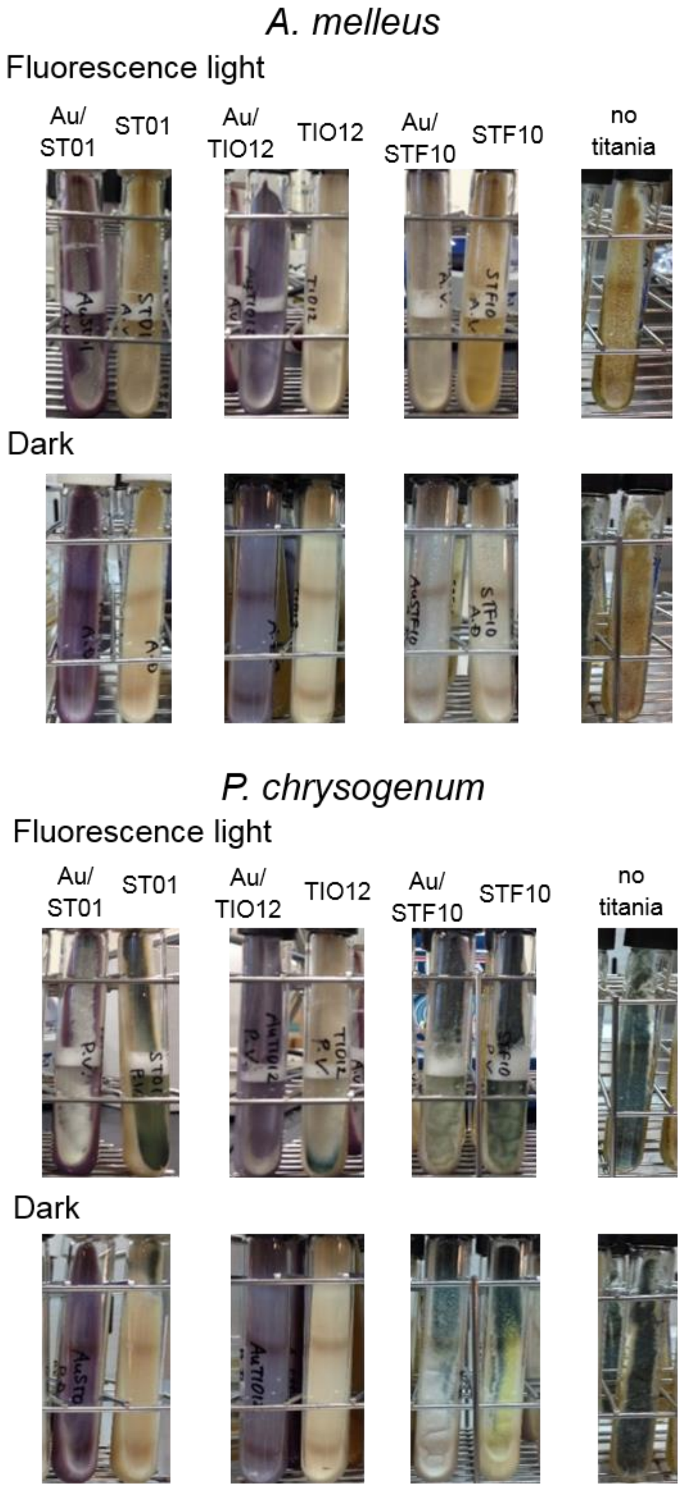

Figure S10: Representative photographs of 5-d growth of *A. melleus* (top) and *P. chrysogenum* (bottom) with Au/TiO_2(ST01), TiO_2(ST01), Au/TiO_2(TIO12), TiO_2(TIO12), Au/TiO_2(STF10), TiO_2(STF10) and no titania samples under fluorescence light irradiation and in the dark.

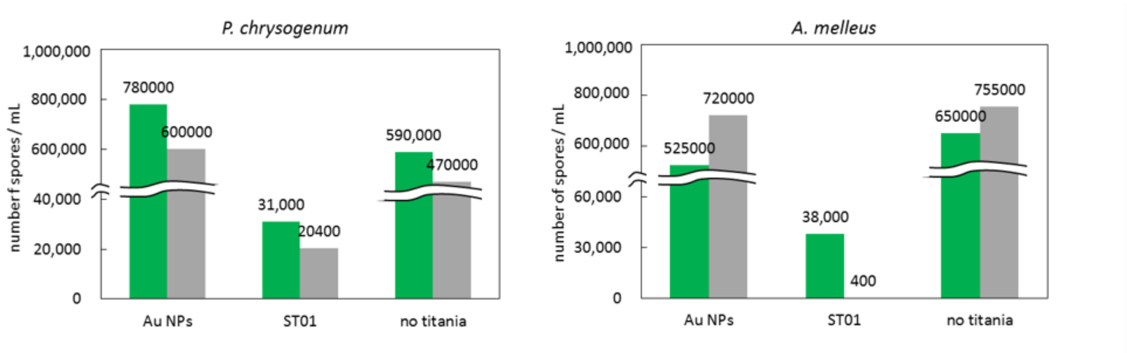

Figure S11: Sporulation of 4-d growth *P. chrysogenum* (left) and *A. melleus* (right) with Au NPs, TiO_2(ST01) and without titania under vis (green) and in the dark (grey).
